# Supplementary material for: Defining the Rhizobium leguminosarum Species Complex
Source: Genes (Basel). 2021 Jan 18;12(1):111. doi: 10.3390/genes12010111 (PMC7831135; doi:10.3390/genes12010111)
Supplement: Supplementary file 1 [file genes-12-00111-s001.zip › Rlc_supplementary_figures.pdf]

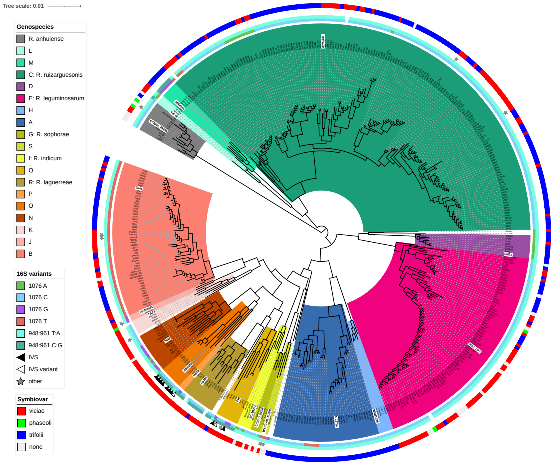

Figure S1. Phylogeny of the Rlc based on 120 core genes using fasttree. The tree is rooted using *R. anhuiense* as the outgroup. The 18 potential genospecies are indicated by coloured segments. Inner circles and symbols indicate variation in the sequence of the 16S rRNA gene (see text for details). The outer circle indicates the symbiovar inferred from the sequence of Nod genes (if any are present in the assembly). The representative strain for each genospecies is highlighted in white. An interactive version (including bootstrap values) is available at https://itol.embl.de/shared/rhizobium.

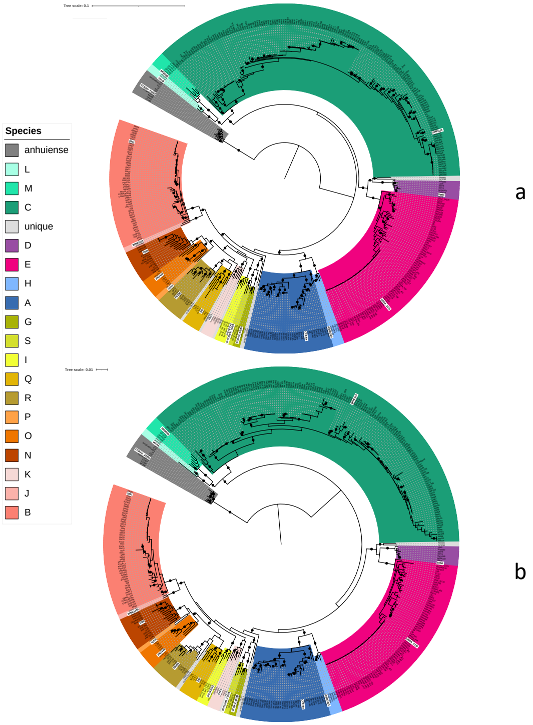

Figure S2. Phylogeny of the Rlc based on two sets of 60 core genes. a. Set A. b. Set B. The trees are rooted using *R. anhuiense* as the outgroup. The genospecies are indicated by coloured segments. Black dots indicate branches with 100% bootstrap support. An interactive version (including bootstrap values) is available at https://itol.embl.de/shared/rhizobium.

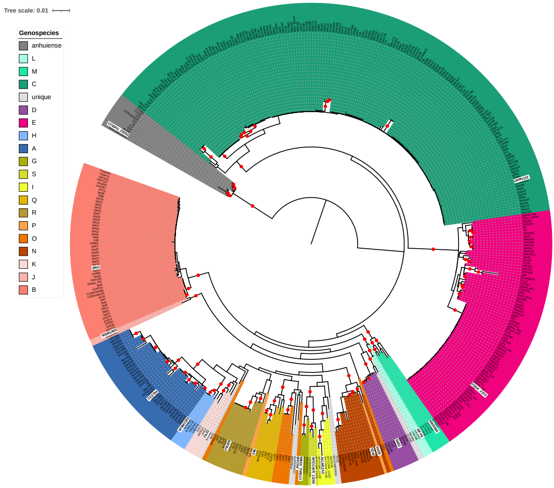

Figure S3. Phylogeny of concatenated *atpD-gyrB-recA* amplicon sequences. The sequences are those that would be amplified by widely-used PCR primers. The genospecies are indicated by coloured segments. Red dots indicate bootstrap support >70%. An interactive version (including bootstrap values) is available at https://itol.embl.de/shared/rhizobium.

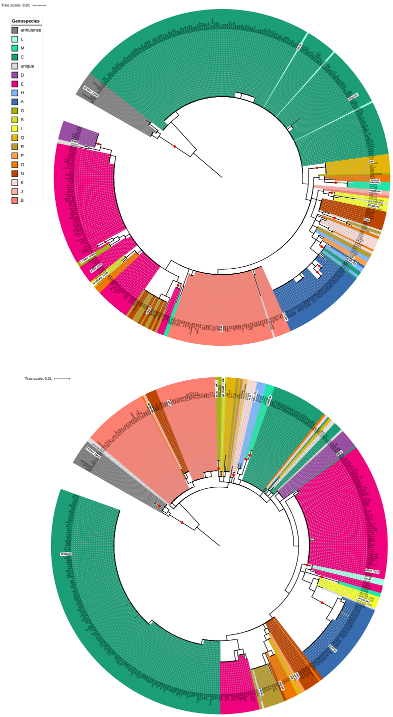

a

b

Figure S4. Phylogeny of two housekeeping genes, based on short sequences that could be amplified for high-throughput sequencing [74]. a. *recA* (251 bp). b. *rpoB* (254 bp). Sectors are shaded to indicate genospecies. Red dots indicate bootstrap support >70%. An interactive version (including bootstrap values) is available at https://itol.embl.de/shared/rhizobium.

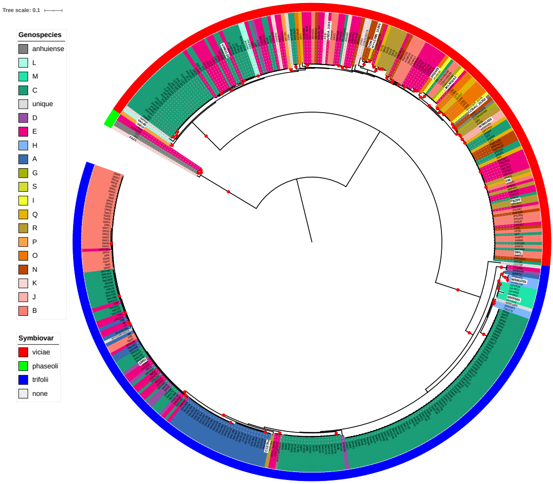

Figure S5. Phylogeny of *nodC*. The outer ring shows the separate clades of the three symbiovars. Sectors are shaded to indicate genospecies. Red dots indicate bootstrap support >70%. An interactive version (including bootstrap values) is available at https://itol.embl.de/shared/rhizobium.

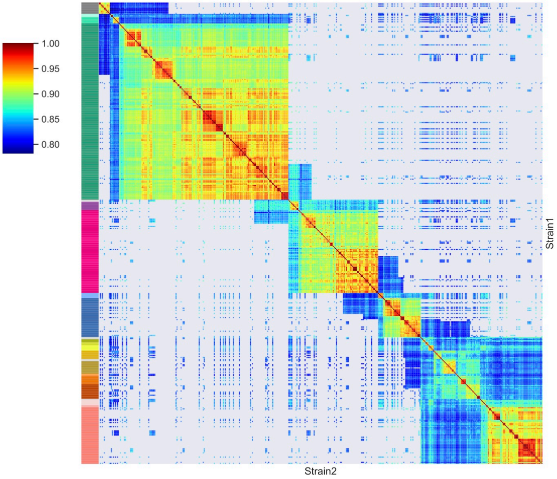

Figure S6. Sharing of accessory genes between strains. Pairwise sharing index, based on the number of orthogroups shared between two strains, normalised by total orthogroup content, for selected comparisons. The genospecies of each strain is indicated in the left bar.
